# Supplementary material for: Advances in Exosomes as Diagnostic and Therapeutic Biomarkers for Gynaecological Malignancies
Source: Cancers (Basel). 2022 Sep 28;14(19):4743. doi: 10.3390/cancers14194743 (PMC9563301; doi:10.3390/cancers14194743)
Supplement: Supplementary file 1 [file cancers-14-04743-s001.zip › cancers-1925930-supplementary.pdf]

Table S1. The relationship between exosomes and gynaecological malignancies.

Disease	Source	Exosomal Cargo	Type	Expression Level	Clinical Value	References
OC	Body fluids	miRNA-205	miRNA	Up	Diagnosis	[19]
		FATS	Protein	Down	Diagnostic and prognostic markers	[20]
		miR-200b	miRNA	Up	Diagnosis and therapeutic target	[21]
		miR-4732-5p	miRNA	Up	Diagnosis and monitoring progress	[22]
	Cells	ANXA2	Protein	-	Metastasis	[24]
		miR-21-5p	miRNA	Up	Progression and therapeutic target	[25]
		lncRNA SOX2-OT	lncRNA	Up	Progression and therapeutic target	[26]
		Circular RNA Foxo3	circRNA	Up	Progression	[27]
		miR-155-5p/PD-L1	miRNA	Down	Inhibiting progression	[28]
		CD47	Protein	Up	Therapeutic target	[29]
		miR-29a-3p	miRNA	-	Progression	[30]
		miR-330-3p	miRNA	-	Therapeutic target	[31]
		circRNA051239	circRNA	Up	Metastasis	[32]
		CD44	Protein	-	Therapeutic target	[33]
		miR-6780b-5p	miRNA	-	Metastasis	[34]
		miR-130a	miRNA	Up	Angiogenesis	[36]
		lncRNA ATB	lncRNA	-	Therapeutic target	[37]
		PKR1	Protein	Down	Angiogenesis	[38]
		miR-92b-3p	miRNA	Down	Anti-angiogenic therapy	[39]
		miR-429	miRNA	Up	Chemoresistance and therapeutic target	[40]
		miR-21-5p	miRNA	-	Chemoresistance and therapeutic target	[41]
		TMEM205	Protein	Up	Chemoresistance and therapeutic target	[44]
		CLPTM1L	Protein	Up	Chemoresistance and therapeutic target	[45]
		miR-484	miRNA	Down	Chemotherapy sensitization	[46]
		miR-497	miRNA	-	Overcome chemoresistance	[74]
CC	Body fluids	lncRNA DLX6-AS1	lncRNA	Up	Diagnosis	[49]
		miR-125a-5p	miRNA	Down	Diagnosis	[50]
	Cells	TIE2	Protein	Up	Angiogenesis	[52]
		Hedgehog-GLI	-	-	Angiogenesis	[53]
		miR-663b	miRNA	Up	Angiogenesis	[54]
		miR-1323	miRNA	Up	Progression and therapeutic target	[55]
		miR-1468-5p	miRNA	Up	Prognostic markers and therapeutic target	[56]
		miR-142-5p	miRNA	Up	Diagnostic marker and therapeutic target	[57]
		miR-663b	miRNA	-	Metastasis	[58]
		lncRNA UCA1	lncRNA	Up	Progression	[59]
		lncRNA AGAP2-AS1	lncRNA	Up	Therapeutic target	[60]
		LINC01305	lncRNA	Up	Progression	[61]
		Wnt2B	Protein	Up	Therapeutic target	[62]
EC	Body fluids	miR-15a-5p	miRNA	Up	Diagnosis	[64]
		LGELS3BP	Protein	Up	Diagnostic and prognostic markers	[65]
		miR-143-3p,miR-195-5p, miR-20b-5p,miR-204-5p, miR-423-3p,miR-484	miRNA	Up	Diagnosis	[66]
		miR-142-3p,miR-146a-5p, miR-151a-5p	miRNA	Up	Diagnosis	[67]
	Cells	lncRNA NEAT1	lncRNA	Up	Therapeutic target	[71]
		hsa_circ_0001610	circRNA	-	Radioresistance	[72]
		miR-26a-5p	miRNA	Down	Metastasis	[73]

OC:ovarian cancer;CC:cervical cancer;EC:Endometrial Cancer
